# Smaller size packs a stronger punch - Recent advances in small antibody fragments targeting tumour-associated carbohydrate antigens

**DOI:** 10.7150/thno.80901

**Published:** 2023-05-15

**Authors:** Sana Khan Khilji, Charlotte Op 't Hoog, David Warschkau, Jost Lühle, Felix Goerdeler, Anika Freitag, Peter H. Seeberger, Oren Moscovitz

**Affiliations:** 1 Department of Biomolecular Systems, Max Planck Institute of Colloids and Interfaces, 14476 Potsdam, Germany.; 2 Institute of Chemistry and Biochemistry, Freie Universität Berlin, 14195 Berlin, Germany.; 3 Graduate School of Life Sciences, Utrecht University, 3584 CH Utrecht, Netherlands.; 4 Institute of Chemistry, University of Potsdam, 14476 Potsdam, Germany.

**Keywords:** fragments, TACAs, cancer, aberrant glycosylation, CAR T-cell, ScFv, nanobody, carbohydrates, tumor-associated carbohydrate antigens, chimeric antigen receptors, single-chain variable fragment, single-chain fragment variable, tumor, Tumour, single domain antibodies, cancer antigens, glycans, immunology, cancer glycans

## Abstract

Attached to proteins, lipids, or forming long, complex chains, glycans represent the most versatile post-translational modification in nature and surround all human cells. Unique glycan structures are monitored by the immune system and differentiate self from non-self and healthy from malignant cells. Aberrant glycosylations, termed tumour-associated carbohydrate antigens (TACAs), are a hallmark of cancer and are correlated with all aspects of cancer biology. Therefore, TACAs represent attractive targets for monoclonal antibodies for cancer diagnosis and therapy. However, due to the thick and dense glycocalyx as well as the tumour micro-environment, conventional antibodies often suffer from restricted access and limited effectiveness *in vivo*. To overcome this issue, many small antibody fragments have come forth, showing similar affinity with better efficiency than their full-length counterparts. Here we review small antibody fragments against specific glycans on tumour cells and highlight their advantages over conventional antibodies.

## Introduction

Glycans are the most complex and structurally diverse biomolecules found on all cells and tissues of the human body. Glycosylation is the primary source of microheterogeneity in proteins, and, with few exceptions, all proteins passing through the ER and Golgi during biosynthesis are modified with *O-* and/or *N-*linked glycans. As part of the cellular glycocalyx, glycans are involved in various biological processes, including intracellular transport, cell adhesion, cell-cell, and cell-matrix interactions, as well as signalling 1. Hence, they play a crucial role in human physiology and pathology.

The assembly of glycans can differ considerably from cell to cell. Although at least 4000 human genes are directly and indirectly involved in glycan synthesis, glycans are not directly encoded in the genome. Genetic defects in the glycosylation machinery often tend to be lethal at the embryonic stage, emphasising the vital role of glycans. The dynamic structure and composition of the glycocalyx are affected by various endogenous factors, such as the expression levels of specific glycosyltransferases and pH in the ER and Golgi. Age and environmental factors, such as lifestyle and diet, have also been shown to affect the glycocalyx. Under normal physiological conditions, a cell's glycan characteristics are primarily conserved, and alterations often reflect and result in cancer 1. These aberrant glycan structures can, therefore, be used as glycan biomarkers for diagnostics as well as specific targeting of the cells that carry them.

Tumour-associated carbohydrate antigens (TACAs) have received increasing attention over the past decades due to their central role in every aspect of cancer progression and the possibility of targeting them specifically using antibodies (Abs). Carbohydrates, however, are generally poorly immunogenic and often elicit a T-cell-independent response, which fails to create immunological memory. Hence, immunisation of animals using glycans primarily leads to the production of low-affinity Immunoglobulin M (IgM), which does not class switch to Immunoglobulin G (IgG) 2. Thus, vaccine development against TACAs is hampered by the absence of T-cell-mediated immunity, which is critical for active cancer immunotherapy. TACAs are often tolerated by the immune system due to the structural similarity of TACAs with healthy cell glycans 3 and the low expression of TACAs in healthy tissue or during prenatal development 4. To overcome the poor immunogenicity of glycans in vaccines and therapeutic Ab development, glycans are conjugated to a carrier protein. The generation of glycopeptides in antigen-presenting cells, following their display by the major histocompatibility complex (MHC) system to T-cell receptors, is essential for eliciting a T-cell-dependent immune response and the formation of specific and high-affinity immunoglobulins. To date, production of most anti-glycan monoclonal antibodies (mAbs) involves animal immunisation with glycan-carrier protein conjugates to ensure the essential T-cell-dependent response for the formation of glycan-specific IgGs.

Even though immunotherapy targeting glycans faces several hurdles, it also holds a considerable advantage. One of the major problems in combating cancer is drug resistance. Due to drug-imposed selection pressure in combination with the high mutational rate and clonal expansion, cancer cells often acquire drug resistance genetically. Targeting TACAs reduces the chance of antigen escape tremendously, as their synthesis, in contrast to proteins, is neither linear nor template-driven and involves the coordinated activity of numerous enzymes. Furthermore, TACAs are far more abundant than protein tumour antigens; for instance, the highly expressed protein marker HER-2 has about 10^6^ copies per cell, whereas the TACA Thomsen-Friedenreich (TF) has about 10^7^ copies per cell 5. TACA expression is often more frequent for given tumour types than protein antigens and identified in a larger percentage of the patients. Targeting TACAs can pave the way to more effective cancer therapies as is demonstrated by the approval of Dinutuximab, a mAb targeting the TACA GD2, for treating neuroblastoma in 2015 6.

**Figure 1:**
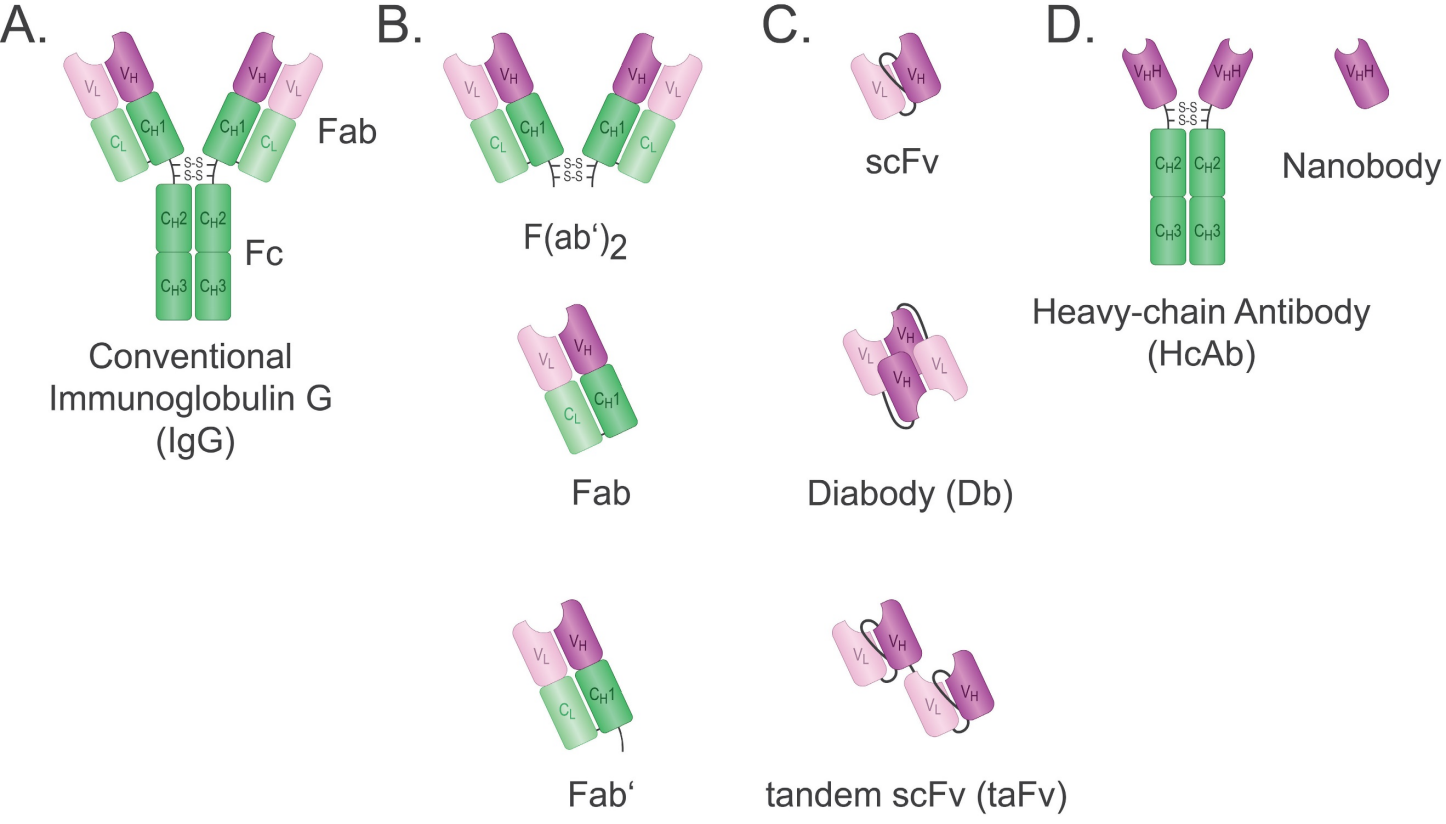
** Antibody and antibody fragments. A.** The full-length Immunoglobulin G (IgG) consists of two Fab and one Fc fragment. The IgG also contains two heavy (H) and two light (L) chains. Green indicates the constant (C) domains and purple indicates the variable (V) domains. The variable domains of the heavy (V_H_) and light (V_L_) chain form the variable fragment (Fv). The Fab and Fc regions are marked. **B.** Fab: Antigen binding fragment; Fab': Fab with hinge region; F(ab')_2_: two Fabs joined at the hinge region. **C.** scFv: single chain variable fragment; diabody: non-covalent scFv dimer; tandem scFv: covalent scFv dimer. **D.** hcAb: heavy chain only Abs from camelids. Nanobody: variable domain (V_HH_) of camelid hcAb.

The mAbs used in therapy are typically IgG and are composed of the fragment antigen binding (Fab), and the crystallizable fragment (Fc) (Figure 1A), which interacts with Fc receptors expressed on several immune cells such as natural killer (NK) cells and macrophages, leading to antibody-dependent cellular cytotoxicity (ADCC) or antibody-dependent cell phagocytosis (ADCP). In addition, the Fc fragment can be recognised by components of the complement system and can thereby induce complement-dependent cytotoxicity (CDC). Alternatively, mAbs can be used to deliver a toxic payload in the form of an antibody-drug conjugate (ADC) 7.

The first mAb for therapeutic use was approved by the U.S Food and Drug Administration (FDA) in 1986. In 2021, the FDA approved the 100^th^ mAb product, highlighting their wide success 8 Nevertheless, their large size and high molecular mass of ~150 kDa, prevents Abs from reaching their full potential in therapeutic efficiency, as penetration into thick and dense tissue, as the tumour microenvironment, is often a limiting factor. In addition, the structure, function, and serum half-life of therapeutic mAbs, as well as non-desired adverse effects in treated human patients are due to Fc fragment interactions with the immune system cells and the N-glycans it carries 9.

To overcome these problems, tremendous efforts have been expended to generate smaller antigen-binding fragments, such as Fab, single chain variable fragment (scFv), and single-domain Abs (Figure 1). Ab fragments are produced through enzymatic cleavage from intact mAbs, or by recombinant protein expression systems such as bacteria, which allow cheaper and faster production in large quantities. The fragments are less immunogenic due to the absence of the Fc fragment, and their size enables better access to dense tissue and contributes to enhanced tumour penetration 10. Moreover, Ab fragments are readily coupled with different molecules, and their relatively smaller size makes them highly amenable to genetic engineering and the development of multivalent and multispecific tools 11**.**

However, many recombinant fragments exhibit reduced stability and affinity compared to their parent Ab. Moreover, lying below the glomerular filtration cut-off of approximately 60 kDa, most Ab fragments suffer from fast renal clearance and a half-life ranging from minutes to several hours 12. A longer circulating time is desirable for cancer therapy to achieve its full therapeutic efficacy through enhanced biodistribution and increased Ab concentration at the target site. Unfortunately, too-small constructs will be excreted through the kidneys before exerting biological effects. Therefore, several strategies are successfully employed to increase the half-life of Ab fragments, including chemical conjugation to polyethylene glycol (PEG) chains or fusion to additional Ab fragments that bind serum proteins such as albumin 13. Nevertheless, a shorter blood circulation time can also be advantageous for tumour imaging, as it lowers background signal compared with the 2-3 weeks of half-life of canonical Abs 14.

While there have been several comprehensive reviews on full Abs targeting TACAs 15-17, literature regarding Ab fragments targeting TACAs was not reviewed to date. Herein, we provide a comprehensive overview of the achievements and applications of Ab fragments that target specific TACAs. First, we provide a structural overview of the different Ab fragments. Thereafter, we describe specific TACAs, and the fragments that target them. Engineered Ab fragments that contain Fc segments are out of the scope of this review.

## Types of small antibody fragments

### Fab-based formats

With a weight of ~50 kDa, the Fab is composed of the Ab light chain (V_L_ + C_L_) covalently bound to the heavy chain V_H_ and C_H1_ domains via a disulfide bridge securing monovalent and monospecific binding (Figure 1B). Fabs are more stable than other fragments such as their single chain variable fragment (scFv) counterparts, but bigger and, therefore, less efficient in tissue penetration 18. Fabs can be obtained readily by proteolytic digestion of IgG with papain. Using alternative enzymatic digestion with pepsin that cleaves below the hinge region, F(ab')_2_ can be obtained. However, this is a time-consuming process as it also requires the production of an Ab first. F(ab')_2_ is a bivalent Ab format with a molecular weight of ~110 kDa. An antivenom F(ab')_2_ is used in the clinic and shows a longer half-life compared to the antivenom Fab previously used 19. No F(ab')_2_ has been approved by the FDA for cancer therapy to date. It is possible to cleave F(ab')_2_ into Fab', which is the Fab containing the hinge region. A major advantage of the various Fabs is that linker engineering is not required, which saves time and resources. Fabs are typically conjugated as targeting ligands for therapeutic or diagnostic tools 20.

### Fv-based formats

#### Single chain variable fragment

The scFv was first described by Bird *et al*. in 1988. It comprises the variable domains of heavy (V_H_) and light (V_L_) chains of an Ab, fused together by a linker peptide (Figure 1C) 21. The linker peptide should be flexible with optimal length and sequence for affinity and thermostability and is often rich in glycine, serine, and hydrophilic residues. The affinity for the antigen is generally comparable to that of the parent Ab 21. While conventional mAbs usually require a mammalian expression system as well as a balance in expression of the light (L) and heavy (H) chains, the V_L_ and V_H_ domains of an scFv are coded in a single genetic sequence leading to approximately 27 kDa that can be easily expressed in both eukaryotic and prokaryotic systems, hence, making it more cost-effective 22. Nevertheless, the disadvantages of scFv should also be taken into consideration. The lack of constant domains often results in lower thermostability and a tendency to aggregate. Thus, scFv production may require additional refolding procedures 23. In addition, scFvs have a short half-life of approximately 0.5-2.0 hours due to their small size. Like Fabs, scFvs are used to generate targeted cancer imaging tracers and drug conjugates 24.

A more recent application of scFvs is their expression as Chimeric Antigen Receptors (CAR) that provide specificity of T cells to their targets but also initiate and determine the strength of their activation 25. CAR-modified immune cells, mainly cytotoxic T-cells but also natural killer (NK) cells, dendritic cells, and macrophages, are genetically engineered to display antigen-specific scFvs or nanobodies. Upon target binding, additional intracellular CAR domains mediate a cytotoxic response and killing of the cancer cell 25. To date, the effectiveness of CAR T therapy against solid tumours remains limited, and the six FDA-approved CAR therapies target different haematological malignancies 26. Nevertheless, extensive efforts are being made to bypass the limitations in targeting solid tumour and generate stable scFv-CARs against protein and glycan targets with optimal affinity to ensure minimal on-target/off-tumour toxicity 25.

#### Multimeric scFv formats: Diabodies and Tandem scFvs

In addition to the classical monovalent form, scFvs can be readily engineered to form multivalent constructs, which is a viable strategy to improve pharmacokinetic properties. The generated multimers are often bivalent, thus having a molecular weight of 50-60 kDa. The increase in size is accompanied by an increase in serum half-life 27. The multivalency can either be utilised to increase the functional affinity, termed avidity, or to target different antigens simultaneously. In case of the latter, the additional binding domain can be used to increase serum half-life further by targeting albumin, or to exert biological effects by targeting effectors. Such bivalent Ab constructs are named bispecific Antibodies (BsAbs). There are two main forms of multimeric scFv formats: diabodies and tandem scFvs (taFv) (Figure 1C). The taFv format is formed by covalent linkage of two scFv molecules through a short linker so that they can rotate freely and retain their separate conformations and antigen-binding units 27. An anti-cancer immune response can be provoked by using an scFv with specificity for cancer cells fused to an scFv with specificity for a type of immune cell. Such a taFv is called a bispecific T-cell engager (BiTE) when a CD3-specific scFv is used to engage T-cells 28.

In diabodies, the two scFvs are not fused together by a linker but instead contain a shorter linker between V_H_ and V_L_ of the same chain forcing the domains to couple with complementary domains of a second scFv. Hence, a rigid conformation is created with two antigen-binding sites. Bispecific diabodies are formed when fusions of variable domains of different Abs (V_H_-V_L_' and V_H_ '-V_L_ or V_L_-V_H_' and V_L_ '-V_H_) as scFvs are expressed in the same cell and interact randomly. A mixture of non-functional scFv-homo- and functional scFv-heterodimers follows, of which the latter can be isolated using affinity chromatography 27. To date, no diabodies are used in the clinic.

### Single domain formats

#### Nanobodies

Three decades ago, the Hamers group at Vrije Universiteit Brussel (VUB) discovered that camelids produce Abs lacking light chains and C_H1_ domains 29. The heavy chain-only antibodies (hcAb) bind to their antigen solely through the two V_HH_ domains that constitute the smallest antigen-binding fragments in nature, weighing only ~15 kDa (Figure 1D). The recombinantly expressed VHH domain was termed nanobody (Nb), and as shaped by nature, Nbs are highly stable, durable, and soluble. Moreover, their small size enables Nbs to penetrate narrow cavities and bind epitopes inaccessible to full-size Abs. Additionally, Nbs are easily genetically manipulated to form multivalent and multispecific tools expressed efficiently on a large scale via different expression systems. Due to their structural and functional advantages, using Nbs and Nb-conjugates for *in vivo* cancer diagnosis and therapeutics is well-studied for a range of intra- and extracellular cancer markers. As a result, five Nb constructs entered (or will soon enter) clinical trials 30, and the first V_HH_-based chimeric antigen receptors (CAR)-T product was FDA approved in February 2022 for the treatment of relapsed or refractory multiple myeloma 31.

In contrast to the long circulation time of full-length mAbs, Nbs exhibit short circulation time and fast renal clearance (> 2h), which leads to reduced background signals and lower accumulation in nontarget organs. Moreover, fast clearance increases the low tumour-to-blood ratio and reduces the systemic side effects often correlated with mAbs. Thus, Nbs were proven ideal reagents for rapid and specific same-day tumour imaging. Nbs advantages over canonical mAbs for tumour imaging were recently demonstrated in several comparison studies in xenografted mice using fluorescently labelled Nbs against HER2 10, 32, and epidermal growth factor receptor (EGFR) 33.

In contrast to mice and humanised mAbs, Nbs are similar to human VH sequences, enabling repeated treatment cycles with a lower risk of anti-drug Ab development 34. Although lacking an F_C_ domain and the ability to engage and activate immune effector functions, Nbs coupled to immunotoxin, drugs, or radio-isotope probes can kill cancer cells, block tumour growth, and prolong the survival time of xenografted mice 11. Moreover, cargo-free Nbs can also inhibit tumour growth by blocking immune-suppressing molecules at the tumour microenvironment or breaking the inhibitory immune checkpoint axis. Nevertheless, in most comparison studies, full-length mAbs with a longer circulation time and the ability to initiate the range of Ab-dependent anti-cancer immune responses still obtain a better anti-cancer therapeutic outcome 35. Yet, despite their obvious limitations for cancer therapy compared to mAbs, several Nbs still demonstrated a comparable, and in some cases, better activity than their full-size counterparts. For example, radioimmunotherapy using anti-CD20 Nbs in hCD20pos B16 tumour-bearing mice significantly prolonged their median survival and was as effective as treatment with the radiolabelled mAb 36. In addition, a unique bispecific nanobody against the immune system suppressing molecules PD-L1 and CXCR4 was superior in inhibiting tumour growth compared with anti-PD-L1 mAb in pancreatic cancer xenografted mice model 37.

Furthermore, due to their structural robustness, Nbs are gaining increased attention as chimeric antigen receptors (CARs) and are investigated in pre-clinical and clinical studies against solid and non-solid tumours 38. Interestingly, in addition to their advantages as CARs, CAR T cells engineered to secrete Nbs in the tumour microenvironment demonstrate enhanced persistence and improved anti-tumoral response compared with conventional CAR T cells 39. Currently, no Nb-based CAR T-cell targeting a TACA has been described. Moreover, obtaining purely anti-glycan Nbs is still challenging 40. Interestingly, we recently reported the development of a specific Nb against Globo-H, a glycosphingolipid expressed in many solid tumours. Using alpaca immunisation with synthetic glycans, we generated a unique Nb that binds specifically to Globo-H, albeit with a relatively low affinity 41.

## Antibody recognition of carbohydrate structures

Our current understanding of how mAbs recognise specific glycan structures has advanced significantly in recent years. It has been found that recognition of particular glycan epitopes by Abs is governed by the precise arrangements of the sugar residues and their linkage, as well as the size and shape of the overall glycan structure. Naturally, higher specificity and lower cross-reactivity of mAbs are dictated by the exact binding epitope along the glycan chain and the number of monosaccharides involved in the interaction. Thus, synthetic glycan array characterisation is crucial to elucidate the specificity and cross-reactivity of anti-glycan Abs. Unfortunately, most existing mAbs cross-react with additional glycan structures, and the number of monospecific anti-glycan Abs is still low. However, currently available three-dimensional structures of Ab-glycan complexes provide valuable information and demonstrate the lack of a universal rule for the binding modes of glycan-targeting Abs and bound glycan conformations. Thus, mAbs recognise distinct glycan epitopes through diverse binding mechanisms. They can exhibit no notable conformational changes upon glycan binding 42 but also undergo an induced-fit conformational change that creates a binding pocket to occupy a portion of the glycan in an end-on insertion 43 or a parallel configuration mode 44. Some mAbs form an extensive glycan-binding paratope that spans the entire V_L_-V_H_ interface, engaging glycans through all six CDRs 45. On the other hand, other Abs rely on only a portion of their CDRs, predominantly those of the V_H_ chain, for glycan binding 46, and some interact exclusively via the V_L_ chain 47. In addition, the bound glycans can adopt an anchored, perpendicular, or parallel orientation to accommodate the Ab binding cavity. Interestingly, recent work on anti-CA19.9 Abs demonstrated that despite the high flexibility of glycan structures, Abs with different binding modes still recognise a similar distinct low-energy conformer 42.

## Targeting tumour-associated carbohydrate antigens

**Figure 2:**
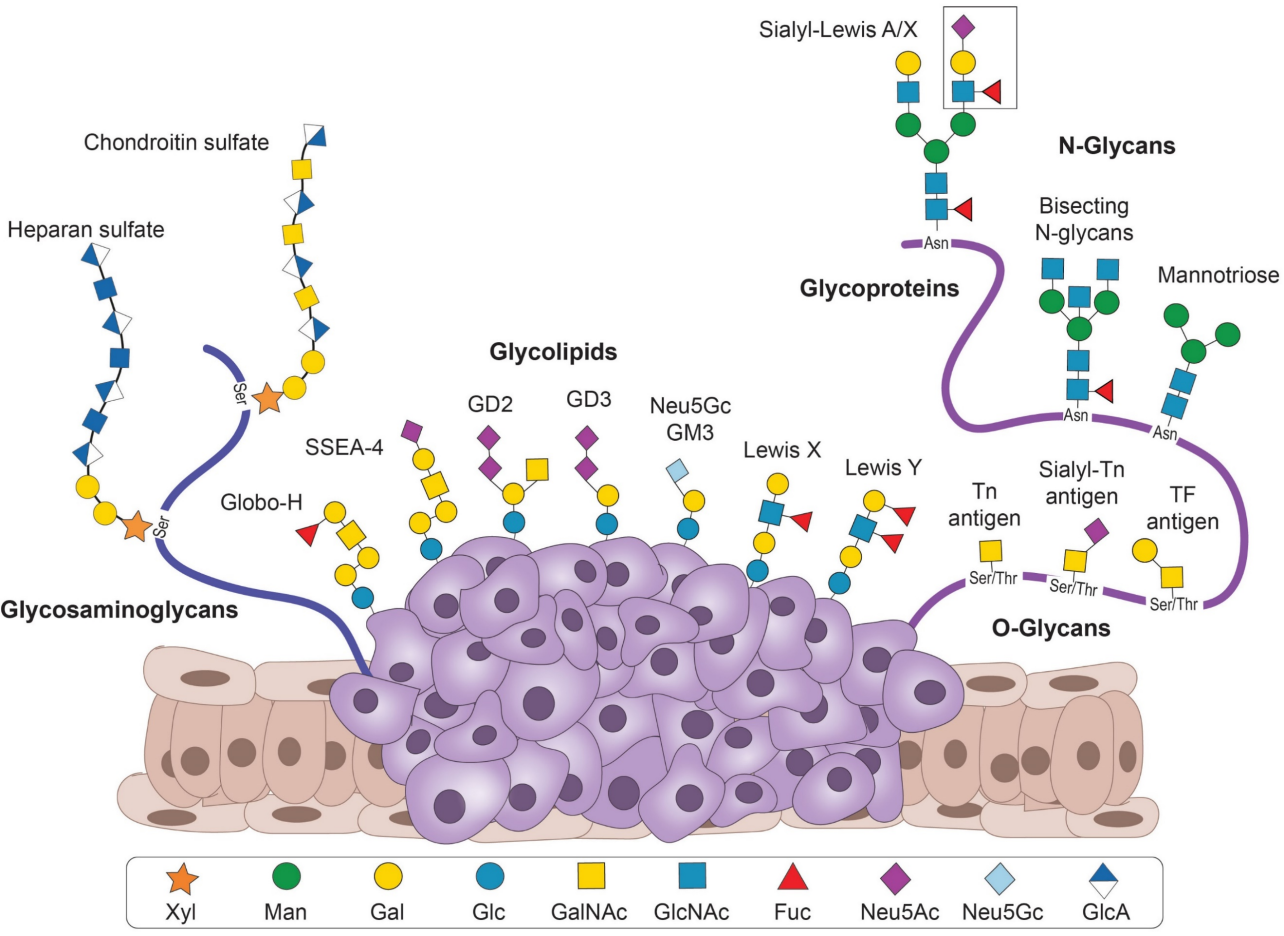
** The structures of the TACAs targeted by small antibody fragments described in this review.** Glycosaminoglycans are shown on the left, TACAs carried by lipids as glycolipids and glycosphingolipids in the middle, and cancer-associated N-and O- glycans carried by proteins (glycoproteins) on the right. Lewis antigens can be present as glycosphingolipids and on glycoproteins.

Small Ab fragments against TACAs have been developed for various applications and target TACAs from several groups (Figure 2). These include 1. Glycosphingolipids from the ganglio-series (GD2, GD3, N-glycolyl GM3), and the globo-series (Globo-H and SSEA-4); 2. Blood group-related Lewis antigens (Lewis Y, Lewis X, sialyl-Lewis X, and sialyl-Lewis A); 3. Aberrant N-glycans or the truncated O-glycans (Thomsen-Freidenreich (TF), Thomsen-nouveau (Tn) and sialyl-Tn (sTn antigens); and 4. several cancer-related glycosaminoglycans. We describe below the antigens, the Ab fragments targeting them, and their applications.

### Glycosphingolipids

Glycosphingolipids (GSLs) are cell surface glycolipids that can be classified according to their core structure as the ganglio-series, the globo-series, and the lacto-series. Each ganglioside series is found in a specific cell type or tissue type and may contribute to adhesion or signalling characteristics of cells 48.

#### Gangliosides

Gangliosides are a GSL subclass, characterised by the presence of one or more sialic acid residues. They interact with phospholipids, cholesterol, and transmembrane proteins and play an essential role in cell adhesion and signalling. Gangliosides are particularly important for the development of the nervous system 49. While most types of gangliosides are widely expressed across tissues, making the majority unsuitable for targeted therapy, some subtypes have limited expression in healthy tissues compared to cancer tissue. Aberrant expression of gangliosides is often detected in tumours of the central or peripheral nervous system (neuroectodermal origin). Aberrant gangliosides that have been targeted using small Ab fragments include GD2, GD3 and N-glycolyl GM3.

##### Disialoganglioside GD2

Disialoganglioside GD2 (GalNAcβ1-4(Neu5Acα2-8Neu5Acα2-3)Galβ1-4Glcβ1Cer) is a TACA found in a wide variety of paediatric and adult malignancies, including neuroblastomas, retinoblastomas, gliomas, osteosarcoma, Ewing sarcoma, rhabdomyosarcoma, small cell lung cancer, melanoma, breast cancer, bladder cancer, and soft tissue sarcoma 50. The U.S. National Cancer Institute ranked GD2 at position 12 out of 75 potential targets based on various aspects such as therapeutic potential, expression level, and specificity 51. GD2 expression in normal tissues is restricted to the central nervous system, peripheral sensory nerves, and skin melanocytes. It presents a promising immunotherapeutic target, especially in case of neuroblastomas, as more than 95% cases of neuroblastomas are GD2-positive. Therefore, mAbs against GD2 have been actively developed in recent years. Currently, the only FDA-approved immunotherapeutic drugs on the market targeting GD2 are Dinutuximab (Unituxin/ch14.18) and Naxitamab (Danyelza/hu3F8), both mouse/human chimeric IgG1 Abs. The Abs are effective in patients, but one of the major side effects is acute pain during administration due to activation of the complement system 52. Therapeutics involving small Ab fragments can overcome this problem due to the absence of Fc-mediated complement activation. Small Ab fragments have been developed from ch14.18 (IgG3), its humanised derivative hu14.18K322A, as well as the class-switched 14G2a (IgG2a). Furthermore, fragments have been generated from hu3F8 (IgG3), 5F11 (IgM), 7A4 (IgG3) and ME361 (IgG2a) Abs. Finally, a Nb targeting GD2 generated by camel immunisation has been recently patented (ID: CN110551218B).

**I. Anti-GD2 Fabs**

The Fabs of 14.G2a, 7A4, and ME361 have been obtained and tested for several applications. Bernhard *et al*. chemically conjugated an anti-CD3 Fab' to ME361 Fab'. The Fab'-based BsAb could specifically mediate lysis of various human GD2-positive melanoma and glioma cell lines *in vitro* by engaging T-cells 53. Later it was shown by Doronin *et al.* that ME361 Fab by itself can inhibit proliferation and induce apoptosis in GD2-expressing mice lymphoma EL4 cells 54. Michon *et al*. chemically conjugated an anti-FcγRI Fab' to 7A4 Fab'. This Fab'-based BsAb (7A4 bis 22) engages macrophages instead of T-cells, which was also successful in binding human neuroblastoma cells *in vitro* as well as grafted in mice 55. The Fab' fragment obtained from 14.G2a was used to produce GD2-targeting liposomes carrying various types of therapeutic agents. Cargo included antisense oligonucleotides against proto-oncogene c-myb, which showed long-term survival (more than 120 days) of mice xenografted with human neuroblastoma 56, chemotherapeutic doxorubicin, which selectively inhibited growth and dissemination of human neuroblastoma in mice 57, and siRNA, which knocked down the gene for anaplastic lymphoma kinase (*ALK*) in neuroblastoma xenografted in mice, causing cell growth arrest, apoptosis, and prolonged survival 58.

**II. Anti-GD2 scFvs**

ScFvs have been generated from 5F11, ch14.18, 14.G2a, hu3F8, KM8138 and 7A4. ScFvs are reported in the application of immunotoxins, pre-targeted radioimmunotherapy (PRIT), BiTEs and CAR T-cells.

Two generated immunotoxins include 14.18-scFv fused to *Pseudomonas* exotoxin A 59 and 5F11-scFv linked to *Diphtheria* toxin A 60 that have shown specific cytotoxicity in neuroblastoma cell lines. However, the immunogenicity of foreign toxins may pose a problem that may be overcome by various approaches such as PEGylation, patient treatment with immunosuppressive drugs, and protein engineering of toxins to modify and/or eliminate immune cell epitopes have been suggested 61.

GD2-targeting scFvs have also been used in radioimmunotherapy. The 5F11-scFv was labelled with streptavidin for multistep delivery of radiolabelled biotin in PRIT 62. First, 5F11-scFv-streptavidin is administered, followed by a chemical clearing agent that removes unbound protein. Subsequently, radiolabelled biotin is added, hence, separating the targeting agent and cytotoxic agent. The tumour-to-blood ratio achieved by this system is almost 60-fold higher in mice compared to radio-iodinated anti-GD2 mAb 3F8 that is currently in phase II clinical trials (NCT00445966). Unfortunately, translation to the clinic is difficult due to the immunogenicity of streptavidin. Nevertheless, a multistep approach employing an scFv to reduce the size and, therefore, clearance time, is promising. Although some efforts have been made to reduce the immunogenicity of streptavidin, other scFv-targeted systems have attracted attention 63.

Incorporating 5F11-scFv into a BiTE can efficiently inhibit melanoma and neuroblastoma xenograft growth 64. A novel platform with human transcription factor HNF1α-derived dimerisation tag enhanced the potency and efficacy of the BiTE by increasing the avidity and serum half-life *in vivo* 65. The hu3F8-scFv has a 13-fold higher affinity (K_D_ = 19 nM) for GD2 compared to 5F11-scFv (K_D_ = 250 nM). Therefore, the group exchanged the anti-GD2 scFv in the construct to assess the effect on the antitumour response. The BiTE holding hu3F8-scFv showed 5000-fold higher potency. This increase in affinity showed improved T-cell activation *in vitro* and better tumour growth inhibition in neuroblastoma and melanoma xenograft models 66.

Hu3F8-scFv has also been engineered to carry a BTX binding peptide that strongly interacts with anti-bungarotoxin (BTX). BTX, on the other hand, was covalently attached to a polymer including doxorubicin 67. While doxorubicin is a popular cytotoxic agent against chemo-sensitive tumours like neuroblastomas, its therapeutic efficacy is limited by dose-dependent toxicity to bone marrow and heart tissue 68. Doxorubicin covalently attached to the scFv directly showed lower binding than the Hu3F8-scFv-BTX-Doxorubicin complex and, therefore, 10x and ~3x lower IC_50_ values against murine lymphoma and human neuroblastoma cells, respectively 67.

Another scFv from murine 3F8 was used to produce GD2-targeted hexanoyl chitosan-based nanoparticles by N-(ß-maleimidopropionyl)succinimide (BMPS) conjugation. The Fab of ME361 was used to compare the BMPS with the carbodiimide (CDI) conjugation method. EL-4 mouse lymphoma cells were used to show that BMPS conjugated constructs exhibited improved binding. The 3F8 scFv showed better binding than ME361 Fab. However, the anti-GD2 fragment-nanoparticle complex was mildly cytotoxic in both cases as cell viability decreased only to about 70% after 72 hours of incubation possibly due to a lack of internalisation of the complex on the cell surface that may be required for cell death induction 69.

The scFv derived from 14.18 was used to generate mono-, di-, and tetra-scFvs by site-directed PEGylation. The di-scFv and tetra-scFv exhibited highest binding efficiency, while tetra-scFvs were better GD2 binders than the di-scFv in a direct enzyme-linked immunosorbent assay (ELISA). The tetra-scFv were also the best binders for the GD2-positive IMR-32 and EL-4 cell lines, while none of the scFv constructs showed binding to the GD2-negative NGP-127 cell line. The cytotoxic effect of tetra-scFv on IMR-32 cells was again higher than the other constructs and comparable to that of the full-length chimeric 14.18 Ab. In addition, in the EL-4 syngeneic mice model, the accumulation of tetra-scFv after 24 h was significantly higher compared to the monomeric scFv and even better than the full-length 14.18 Ab. This highlights the significance and advantage of multimerisation of small Ab fragments for better therapeutic characteristics 70.

More than two decades ago, the 7A4 Ab was used to generate an scFv. The binding was tested in an ELISA and cross-reactivity with other closely related gangliosides was included. ScFv 7A4 retains its target specificity with weak cross-reactivity to GD3, as is seen for the full Ab 71. Later, scFv 7A4 was incorporated into a CAR and expressed in T-cells. The CAR T-cells were, however, not functional. In the same report, scFv 14.G2a was also tested, which showed neuroblastoma-specific cell lysis. However, functionality decreased over time without nonspecific stimulation of transduced cells, and antigenic stimulation of the chimeric receptor alone could not sustain proliferation 72. Many reports followed this GD2-targeting CAR T-cell since. Sujjitjoon *et al.* generated T-cells with fourth generation CAR moieties. For this, the scFv derived from mAb hu3F8 was used, and GD2 expressed on retinoblastomas was targeted. The CAR T-cells were effective in killing retinoblastomas, but extended exposure to the T-cells diminished their effect on the retinoblastomas. Immune evasion mechanism by retinoblastomas was also studied. This was the first study to show a CAR T-cell strategy targeting retinoblastomas 73.

The expression of TACAs on healthy tissues, albeit low, can cause off-target effects in conventional CAR T-cell therapy. CAR T-cells targeting large tumours may unpredictably proliferate and cause adverse effects like cytokine storms and tumour lysis syndrome in patients. Mitwasi *et al.* used a novel CAR T-cell (UniCAR) approach to target GD2. Unlike conventional CAR T-cells, UniCAR T-cells are not targeted towards a cancer cell surface epitope directly. Instead, they bind to a peptide epitope (so-called target module (TM)) that is not only specific for the cancer cell surface epitope but also modulates the UniCAR T-cells in a switch on/off manner as the UniCAR T-cells would target the cancer cell only in their presence. In this case, the TM comprised the scFv 14.G2a fused with the UniCAR epitope. Three GD2-specific TMs were used to show target-specific and -dependent activation of UniCAR T-cells, secretion of pro-inflammatory cytokines, and tumour cell lysis for Ewing sarcoma and neuroblastoma *in vitro*. TM enrichment and rapid elimination at the tumour site were studied via PET imaging *in vivo* in a neuroblastoma mouse model to confirm the TMs fulfilled all prerequisites 74. As a proof of concept, the same group used NK-92 cells as an alternative to the CAR T-cells with the same UniCAR strategy to target GD2 on neuroblastomas *in vitro* and *in vivo.* NK cells boast a lower risk of toxicities. In addition to using scFv 14.G2a fused with the UniCAR epitope, they studied varying half-life of TMs, by also generating an IgG4-Fc-fused scFv 14.G2a and UniCAR epitope. Specific killing of GD2-expressing cells *in vitro* and *in vivo* was observed, along with increased production of interferon-γ. With the help of radiolabelled proteins, it was demonstrated that the IgG4 Fc region and homodimerisation of the TM molecule increased the *in vivo* half-life dramatically 75.

Previously, first-generation CAR NK92 cells were also generated using scFv ch14.18 targeting GD2 on neuroblastoma, melanoma, and breast cancer cells. Specific targeting and cytotoxicity against neuroblastoma cells initially resistant to parental NK-92 was established. Targeting was blocked by GD2-specific mAb or an anti-idiotypic mAb occupying the CAR's cell recognition domain. Additionally, enhanced cytotoxicity of CAR NK-92 targeting GD2 was found against primary neuroblastoma cells as well as GD2-expressing melanoma and breast cancer cells, offering potential clinical utility of retargeted effector cells 76. Several GD2-targeting CAR T-cells reached clinical trials and the results of three phase I clinical trials for children with neuroblastoma were published. First-generation CAR T-cells comprising autologous activated T-cells and Epstein Barr-virus specific T-cells were both modified to express a CAR construct containing the scFv 14.G2a (NCT00085930). The treatment did not induce neurotoxicity and lead to complete remission in 3/19 patients, which has persisted for 2 patients for at least four years 77. A third generation CAR T containing scFv 14.G2a and an inducible caspase 9 suicide switch was tested in neuroblastoma patients (NCT01822652). Patients were divided in three cohorts: CAR T alone, CAR T with preparative lymphodepletion and CAR T with lymphodepletion and a PD-1 inhibitor. The lymphodepletion resulted in increased CAR T expansion. PD-1 inhibition did, however, not result in enhanced expansion or persistence. The treatments were safe and lead to a modest antitumour activity after six weeks 78.

1RG-CART, a second-generation CAR with scFv KM8138 was tested in children with relapsed/refractory neuroblastoma (NCT02761915). Three out of six patients receiving high doses (≥10^8^/m^2^ CAR T-cells) in combination with preparative lymphodepletion showed antitumour activity in sites of disease, without on-target off-tumour neurotoxicity. Nonetheless, the effect was transient and modifications to enhance T-cell persistence are required 79. In another study, the fourth generation 4SCAR-GD2 containing the scFv hu3F8 and an inducible caspase 9 suicide switch was tested (NCT02765243). Tumour growth was delayed, and patients' survival was prolonged without neurotoxicity. Nevertheless, the 4SCAR-GD2 T-cells expanded when the disease relapsed, indicating other factors at play determining treatment efficacy 80. Clinical trials based on GD2-targeting CAR T-cells are listed in Table 1.

##### Disialoganglioside GD3

Another highly relevant ganglioside TACA is GD3 (Neu5Acα2-8Neu5Acα2-3Galβ1-4Glcβ1Cer), which, like GD2, is found in cancers of neuroectodermal origin 81. Its normal expression pattern is mainly restricted to high levels in embryonic neural stem cells 82 and low levels in melanocytes, retinal pigment cells and the central nervous system (CNS) 83, 84. GD3 is highly overexpressed in melanoma, astrocytoma, medulloblastoma, meningioma, neuroblastoma, lung, and breast cancer, while 9-O-acetyl-GD3 is overexpressed in lymphoblasts of childhood acute lymphoblastic leukaemia 81. The use of a small Ab fragment targeting GD3 for cancer therapy is limited to the application of the scFv from MB3.6 murine mAb in CAR. A first-generation CAR 85 and later a second-generation CAR was created 86. Following treatment with the second-generation CAR in combination with IL-2, a remission rate of 50% was observed in a subcutaneous melanoma mouse model. In addition, the CAR T-cells could substantially reduce the tumour burden in a genetic mouse model of tuberous sclerosis complex (TSC). TSC is a genetic disease that causes benign tumours to grow. The tumours mostly affect the brain, skin, kidneys, heart, and lungs 87. To date, there is no cure for TSC, rendering anti-GD3 CAR T a valuable therapeutic option.

Kotlan *et al*. reported a method that can aid in biomarker identification and simultaneously generate human-derived scFvs against an antigen. Tumour infiltrating B (TIL-B) cells were isolated from metastatic melanoma, and following RT-PCR, the variable domains were cloned in the scFv format to generate an scFv library. Following phage display and binding evaluation to melanoma cells, binders were found for GD3 and its *O*-acetylated form. The presented method can provide means to obtain tumour-specific Abs of human origin. The study provides evidence for a two-way regulation mechanism between TIL-B cells and tumour cells and opens a way to potentially new cancer treatment strategies 88.

##### N-Glycolylneuraminic acid GM3

The N-Acetylneuraminic acid (NeuAcGM3) ganglioside is normally detected in normal human tissues. However, N-glycolylneuraminic acid (NeuGcGM3) ganglioside is a TACA found in various tumours, including lung cancer, breast cancer, neuroblastoma, pancreatic cancer, and gastrointestinal tumour 89. As healthy cells are incapable of synthesising Neu5Gc due to a deletion in the CMP-NeuAc hydroxylase gene, the current hypothesis to explain the presence of NeuGcGM3 on malignant cells is attributed to incorporation from dietary sources due to altered metabolism and hypoxia 89. Due to the low expression in normal tissue, NeuGcGM3 is an attractive therapeutic target candidate. However, a close structural homologue, N-acetyl GM3 is widely expressed in the body and only differs from N-glycolyl GM3 by a single oxygen atom. The murine mAb 14F7 targets NeuGcGM3 specifically with no cross-reactivity with NeuAcGM3 90. Therefore, Roque-Navarro et al. generated Fabs of 14F7 and showed, that while 14F7-Fab does not induce cytotoxicity in murine lymphocytic leukaemia, F(ab')2 retains the induction capacity of the full-length Ab, possibly due to cross-linking of carbohydrate epitopes. The novel mechanism of NeuGcGM3-mediated 14F7-induced cell death accompanied by cellular swelling, membrane lesion formation, and cytoskeleton activation, suggested a complement-independent phenomenon, and demonstrated the therapeutic usefulness of NeuGcGM3 for Ab-based cancer immunotherapy 91. The generation of an scFv from 14F7 by fusing the original hybridoma variable regions did not yield a functional Ab fragment. Light-chain shuffling trying various combinations of the original 14F7 VH region with various naïve VL regions followed by phage display yielded various scFvs with affinities ranging from KD = 15-38 nM, thus retaining the affinity and specificity of 14F7 (KD = 25±4 nM) 92.

#### Globo family

The Globo family shares the core structure Galα1-4Galβ1-4Glcβ-Cer, and the TACAs in this family include Stage Specific Antigen 3 ((SSEA-3) also called GB5), Globo-H (SSEA-3b), and SSEA-4. The last two have received the greatest attention as candidate targets. Globo-H and SSEA-4 are both synthesised from SSEA-3, and differ only by their terminal moiety, which is fucose or sialic acid, respectively 93.

##### Globo-H

Globo-H (Fucα1-2Galβ1-3GalNAcβ1-3Galα1-4Galβ1-4Glcβ1-Cer) is overexpressed in a wide variety of epithelial cancers, including breast, gastric, lung, ovarian, endometrial, pancreatic, and prostate cancers. Globo-H has been shown to promote immunosuppression, angiogenesis, and metastasis of tumours 15. During normal development, Globo-H is abundantly expressed in undifferentiated embryonic stem cells (ESCs) and is lost after differentiation 94. Later, in normal differentiated tissue, it is only moderately expressed in apical epithelial cells at the lumen borders of glandular tissue, which are inaccessible to the immune system 15. Hence, Globo-H is a promising candidate for immunotherapies. Most research regarding Globo-H therapeutics is focused on the development of active immunotherapy, however, one mAb targeting Globo-H, OBI-888, is currently being tested in phase I/II clinical trials for locally advanced or metastatic solid tumours with sufficient target expression (NCT03573544). In addition, OBI-999, an ADC composed of a Globo-H-specific mAb and the anti-mitotic agent monomethyl auristatin E, is also being tested in phase I/II clinical trials (NCT04084366). So far, only one small Ab fragment was described against Globo-H, a Nb termed GH46 that we produced through immunisation of an alpaca with synthetic Globo-H conjugated to a carrier protein 41. Although highly specific to its target, the monovalent GH46 exhibited a relatively low affinity to Globo-H. Nevertheless, a trivalent construct of the Nb showed a nine-fold improved affinity (apparent K_D_ from 18 ± 8 μM to 2 ± 1 μM) through improved avidity on breast cancer cells 41.

##### SSEA-4

Like Globo-H, SSEA-4 (Siaα2-3Galβ1-3GalNAcβ1-3Galα1-4Galβ14Glcβ1-1Cer) is expressed in ESCs. Previously, it was believed that the expression is strictly limited to the embryonic stage, and that expression in adults is limited to malignant tissues. Several examples of such malignancies include malignant glioma cells, breast cancer, ovarian cancer, teratocarcinoma, lung cancer, and prostate cancer. In cancer cells, SSEA-4 promotes invasion and metastasis by mediating loss of the cell-cell interactions and the gain of a migratory phenotype. SSEA-4-expressing cancer cells often display high levels of stem cell-specific markers 93. Therefore, SSEA-4 seemed an excellent TACA to target for anti-cancer therapy. However, SSEA-4 is in fact expressed in healthy adult tissues, namely in the mesenchymal stem cell population in bone marrow 95 and in germ cells in testis 96, and ovary 97. Probably owing to this, so far, no agents targeting SSEA-4 have entered clinical trials. Nevertheless, efforts are being made towards developing SSEA-4-targeted CAR T-cell therapy. Pfeifer *et al*. generated a second-generation CAR using an scFv derived from the SSEA-4-specific mAb REA101 (Miltenyi Biotec). The CAR T-cells could inhibit triple-negative breast cancer cells *in vitro* and *in vivo* in a xenograft mouse model. However, on-target/off-tumour toxicity was observed, due to the co-targeting of hematopoietic multipotent progenitor cells in the bone marrow and pluripotent epithelial cells in the lungs expressing SSEA-4 98, 99. On the other hand, Lin *et al*. generated a second-generation CAR with the scFv of a newly developed humanised murine mAb hMC48. The CAR T-cells could inhibit pancreatic cancer cells *in vitro* and *in vivo* in a xenograft mouse model without inducing any toxicity 100.

### Lewis blood antigens

The blood type is determined by the specific oligosaccharide structures found on various cells, such as red blood cells, platelets, leucocytes, plasma proteins, certain tissues, and various cell surface enzymes. These glycans also exist in soluble form in body secretions such as breast milk, seminal fluid, saliva, sweat, gastric secretions, urine as well as amniotic fluid, and their composition is defined by the expression of specific glycosyltransferase enzymes. Since the discovery of blood antigens in the early 20^th^ century, over 30 blood group systems have been described 101. One of these systems concerns the Lewis blood antigens. The Lewis antigens can be carried on GSLs, as well as *N*- and *O*-glycans 102. The antigens are primarily involved in cell adhesion and cell signalling during embryogenesis and later development. Aberrant expression of Lewis antigens correlates with malignant transformation and poor prognosis. Therefore, they constitute an attractive class of TACAs. The Lewis antigens are all structurally related, in which three monosaccharide units are present: *N*-acetylglucosamine, galactose, and fucose. The different glycosidic bonds can give rise to different Lewis antigens (Galβ1-3GlcNAc in type I and Galβ1-4GlcNAc in type II Lewis antigens). The TACAs that have been targeted using small Ab fragments include Lewis Y (Le^Y^) and Lewis X (Le^X^), as well as the sialylated Lewis antigens sialyl-Lewis A (sLe^A^) and sialyl-Lewis X (sLe^X^) 103.

#### Lewis Y

Le^Y^ (CD174) is a difucosylated tetrasaccharide (Fucα1-2Galβ1-4Fucα1 -3GlcNAc). In healthy tissue, Le^Y^ is mainly found in several epithelial tissues and weakly on granulocytes. Overexpression of Le^Y^ has been reported for breast, gastrointestinal, pancreatic, ovarian, and prostate cancers, as well as hepatocellular carcinoma, and non-small-cell lung carcinoma 103. In addition, the upregulated expression of Le^Y^ on endothelial cells contributes to tumour vascularisation and leucocyte recruitment to inflamed tissue 104.

##### I. Lewis Y-targeting immunotoxins

Since the early 1990s, efforts have been made to generate Le^Y^-targeting therapies using small Ab fragments. The older therapeutics that were primarily developed were immunotoxins. The first immunotoxin targeting Le^Y^ using a small Ab fragment is LMB-7. It comprises of an scFv derived from the murine mAb B3 and a truncated form of *Pseudomonas* exotoxin A, which inhibits protein synthesis. Later, a disulfide stabilised version was generated and was called LMB-9. Both immunotoxins showed a binding affinity of K_D_ = ~1.5 nM 105 and were tested in phase I clinical trials. LMB-7 was tested in patients with leptomeningeal metastases (NCT00003020) and LMB-9 in patients with advanced colon, breast, non-small cell lung, bladder, pancreatic, or ovarian cancer (NCT00005858). Patients experienced renal and gastrointestinal toxicities, due to the expression of Le^Y^ on normal gastric cells and some tubular cells in the kidney. The latter could be prevented by blocking acid secretion in the stomach, however renal toxicity remained dose limiting. Therefore, no significant anti-tumour activity could be observed 106.

A similar immunotoxin was generated by conjugating a truncated *Pseudomonas* exotoxin to Ab fragments derived from the mouse-human chimeric IgG BR96. First, the Fab formats (Fab', and F(ab')_2_) were compared to the full-length IgG immunotoxin conjugates. Although internalisation was similar, the Fab' immunotoxin was considerably less cytotoxic than F(ab')_2_ and IgG. This was believed to be a result of loss of avidity through the monovalent nature of Fab' 107. Nevertheless, later the toxin was genetically fused to an scFv derived from BR96 such that the construct was specific for Le^Y^, although with 5-fold less binding compared to the mAb BR96. Interestingly, the scFv-based toxin was 4-fold more potent than the intact IgG-immunotoxin construct on Le^Y^-positive MCF-7 cells 108. The scFv immunotoxin is approximately one-third of the size of the IgG immunotoxin, resulting in four times shorter blood half-life time and enhanced tumour penetration in breast and lung xenograft mice models 109. BR96 scFv-PE40 requires no chemical conjugation and can be produced entirely in bacteria relatively rapidly and inexpensively 108. BR96 scFv-PE40 was named SGN-10 and entered phase I clinical trials for patients with different types of Le^Y^-positive metastatic carcinomas. About one-third of the patients showed no signs of disease progression, indicating a favourable outcome. However, the treatment was hampered by human antitoxin Ab (HATA) and toxicity responses due to the expression of Le^Y^ on gastrointestinal epithelial cells 110.

Harmful HATA responses can be bypassed using human toxins. A generated immunotoxin (K_D_ = ~0.45 µM) composed of an scFv derived from the Le^Y^-specific mAb B1 and tumour necrosis factor α (TNF-α), a protein that can induce cell death upon binding to cell surface receptors, was specifically cytotoxic *in vitro* and *in vivo* in TNF-α-sensitive MCF-7 human breast cancer cells xenograft mice 111.

##### II. Lewis Y-specific multimers

To generate enhanced anti-Le^Y^ fragments, Rheinnecker *et al.* developed an scFv derived from MSL5 (murine anti-Le^Y^ IgM). Self-assembling multimers were generated by fusing the scFv sequence to either an artificial dimerisation domain or the tetramerisation domain of the human transcription factor p53. The monomeric scFv showed specific but weak binding to Le^Y^ on surface plasmon resonance (SPR). In contrast, the dimeric and the tetrameric scFv exhibited an eight-fold and a twenty-fold increase in binding through increased avidity 112. Power *et al.* generated a diabody against Le^Y^ by altering the linker length between V_H_ and V_L_ in an scFv. By using a five-residue linker in the scFv derived from humanised mAb hu3S193, the generated construct was pushed to form a non-covalent bivalent diabody. The diabody with a molecular weight of 54 kDa demonstrated similar binding activity as the parent mAb on SPR as well as in MCF-7 cell binding assays. After radiolabelling the diabody, rapid tumour uptake and fast blood clearance were observed in an MCF-7 xenograft mouse model. The Le^Y^-specific diabody proved to be a promising candidate for tumour imaging 113. The ^111^In-radiolabelled C-functionalised trans-cyclohexyl diethylenetriaminepentaacetic acid chelated diabody was compared to F(ab')_2_ (generated with pepsin digest of mAb hu3S193) in terms of affinity and biodistribution in breast carcinoma xenograft mice. The affinity of the diabody was approximately 10-fold lower than the intact mAb. The F(ab′)_2_ displayed a somewhat higher affinity than the diabody, as well as increased stability. The results demonstrate that the diabody is more effective as a diagnostic imaging tool, whereas F(ab')_2_ is better for tumour targeting 114. Obtaining multimers by varying the linker length was explored to generate other multimers. The scFvs a linker that is extended by three residues length formed scFv dimers, whereas decreasing the length or completely removing the linker formed scFv triabodies and tetrabodies. Expressed in *Escherichia coli*, all scFv formats gave active protein 115. The affinity of the hu3S193 multimers expressed as K_a_ was 158 nM, which is slightly higher, compared to hu3S193 mAb. On the other hand, the affinity of hu3S193 F(ab′)_2_ was 4.31 µM, which is approximately 10-fold lower. Further experiments showed maximum tumour uptake for the scFv trimer/tetramer mixture within six hours and for the F(ab')_2_ within 24 hours in MCF-7 xenografted mice. Despite the tumour targeting, however, high *in vitro* instability and renal uptake prevented the scFv multimer mixture from being used in radioimmunotherapy and imaging 116.

##### III. Lewis Y-targeting CAR T-cells

Targeting Le^Y^ with CAR T-cells was first described for CAR targeting Le^Y^ used an scFv derived from the MluC1 mAb as antigen-binding domain. The scFv was linked to the γ-chain of the Fc receptor and transduced to cytotoxic T-cells. Even though the binding ability of the soluble scFv was low, seven of thirteen genetically engineered cytotoxic T lymphocyte clones inhibited the growth of Le^Y^-positive cells *in vitro* 117. More advanced, second-generation CAR T-cells were generated using the scFv derived for the first time from a humanised mAb, hu3S193, targeting Le^Y^. The engineered T-cells inhibited subcutaneous human ovarian OVCAR-3 tumours, which are recognised as a difficult model to treat, in xenograft mice. Normal tissue cells expressing lower amounts of antigen were not harmed by T-cells that were redirected towards them. The antitumour response was not inhibited in presence of excess numbers of normal Le^Y+^ cells 118. Based on these findings, the tolerability and potential therapeutic effect of anti-Le^Y^ CAR T-cells were examined in a phase I clinical trial with four patients suffering from acute myeloid leukaemia. While CAR T-cell infusions were generally tolerated well and the CAR T-cells accumulated and persisted in the bone marrow and the spleen, they failed to impair tumour progression in patients 119. Since Le^Y^ has generally higher expression on epithelial tumours, another phase I clinical study is currently assessing the tolerability and potential anti-tumour activity of anti-Le^Y^ CAR T-cells in patients with advanced solid tumours (NCT03851146).

#### Lewis X and sialyl-Lewis X

Le^X^ (SSEA-1/CD15) is a trisaccharide (Galβ1-4(Fucα1-3)GlcNAc) that is involved in embryonic development, particularly of the central nervous system (CNS). In addition, it is a typical antigen found on myeloid cells, while also expressed in normal tissues of the stomach, colon, salivary glands, kidneys, bladder, epididymis, uterus, cervix, and medulla, and weakly in several other tissues. Le^X^ is overexpressed on various types of cancer cells, including both haematological malignancies (Hodgkin's lymphoma) and solid tumours (colorectal, thyroid, urological, lung, breast, and oral cancer, as well as hepatocellular carcinoma and glioma). Moreover, it is a ligand for selectins and thereby mediates adhesion to endothelial cells and transendothelial migration. Overexpression of Le^X^ on cancer cells has a pivotal role in metastasis 103.

Sialylation of Le^X^ forms the tetrasaccharide sLe^X^ (NeuAc-Galβ1-4(Fucα1-3)GlcNAc). sLe^X^ is expressed on neutrophils and monocytes, allowing for extravasation to sites of inflammation via their interaction with selectins 103. In contrast to Le^X^, sLe^X^ can also be present in serum in soluble form, where it can compete for interactions with selectins and is, therefore, correlated with favourable prognosis 120. However, overexpression on the cancer cell surface is correlated with poor prognosis. sLe^X^ overexpression has been reported in gastrointestinal, bladder, prostate, lung, and breast cancer 103.

ScFvs are generally obtained from murine sources. To obtain completely human scFvs targeting Le^X^ and sLe^X^, a phage-display library derived from the peripheral blood lymphocytes of 20 patients of various cancer types to select and isolate scFvs specific to the carbohydrate antigens was generated. At least four different scFv genes could be obtained that have K_D_ values ranging from 0.11 nM to 0.62 nM, which are comparable to the affinities of the mAbs. The isolated scFvs showed specificity for Le^X^ and sLe^X^ on pancreatic adenocarcinoma cells. This was the first time the technique was employed to generate human Ab fragments against TACAs directly 121. In another study, the two human scFvs, LeX1 and sLeX10, selected from the n-CoDeR phage display library and targeting Le^X^ and sLe^X^, respectively, were shown to bind rapidly and specifically to their antigens on SPR. LeX1 and sLeX10 showed relatively low K_D_ values of 35 ± 7 μM for Le^X^ and 26 ± 7 μM for sLe^X^, respectively 122.

#### Sialyl-Lewis A

sLe^A^ (CA19.9/CD43) is a tetrasaccharide (Neu5Acα2-3Galβ1-3(Fucα1-4)GlcNAc) that is normally expressed in low levels on fibroblasts and on luminal and glandular epithelium 103. However, sLe^A^ expression is particularly elevated in gastrointestinal cancers. Notably, sLe^A^ is the only FDA-approved biomarker for pancreatic cancer 123. Disialyl-Lewis A (Le^A^), sLe^A^ with an additional sialic acid, is commonly expressed on non-malignant epithelial cells and is a ligand for immunosuppressive receptors. During malignant transformation, the overexpression of sLe^A^ originates from the incomplete synthesis of Le^A^. Hence, the aberrant expression contributes to cancer progression in two ways: the loss of Le^A^ contributes to tumour-promoting inflammation, and gain of sLe^A^ allows binding to selectins, like Le^X^ and sLe^X^ 124.

F(ab')_2_ prepared from the murine SA23.2 IgM mAb bound more strongly to sLe^A^-positive colon adenocarcinomas, pancreatic, stomach, and lung cancers, and showed much less unspecific binding than the original IgM 125. As a follow-up, BsF(ab')_2_ from SA23.2 IgM and anti-carcinoembryonic antigen (CEA) IgM was generated via disulfide bond exchange. The BsF(ab')_2_ had almost the same affinity (K_D_ = 0.17 μM) as the parent F(ab')_2_ construct (K_D_ = 0.18 μM). While the size and low solubility of an IgM restrict its number of applications, the BsF(ab')_2_ format might be a useful immunotherapy and -diagnosis tool 126. A radiolabelled diabody from the murine mAb 1116-NS-19-9, with an apparent affinity of K_D_ < 3 nM, can be conjugated to nanoparticles and was suitable for PET imaging in a pancreatic cell xenograft mouse model 127. The diabody was conjugated to liposomal nanoparticles that specifically target sLe^A^-expressing pancreatic cells *in vitro* as well as xenografted in mice, indicating the potential of the diabody to deliver targeted treatment 128. Human 5B1 (MVT-5873), another mAb against sLe^A^ is currently being tested in phase I clinical trials in patients with advanced pancreatic cancer or other sLe^A^ positive malignancies (NCT02672917). Borenstein-Katz *et al*. used sequences of both mAbs, 5B1 and 1116-NS-19-9, to generate scFvs using yeast surface display. These were used in return to generate a mutated version of 1116-NS-19-9 with 10-fold increased affinity and computer-assisted sequence optimisation 42. An scFv derived from 5B1 was used to develop a sLe^A^-targeting CAR T-cell therapy. The 5B1-based CAR T-cells were tested *in vivo* in an orthotopic pancreatic cancer mouse model in combination with low-dose radiation. The radiation was used to sensitise cancer cells to activated CAR T-cells. Remarkably, sLe^A-^ cancer cells were also eliminated upon immune activation by sLe^A+^ cancer cells. Thus, the therapy is promising for targeting heterogenous solid tumours 129.

### Glycoproteins

#### Truncated *O-*glycans

Aberrant *O*-glycan structures in mucins and other glycoproteins are abundantly found in many carcinomas and associated with cancer aggressiveness as well as poor prognosis. Their expression directly affects oncogenic parameters such as cancer cell proliferation, motility, and invasion. *O-*glycan TACAs include the Thomsen-nouveau antigen (Tn or CD175, GalNAcα-*O*-Ser/Thr) and its sialylated structure sTn (CD175s/CA72-4), as well as Thomsen-Friedenreich antigen (TF/T antigen or CD176, Galβ1-3GalNAcα-*O*-Ser/Thr) with its mono- and di-sialylated forms 130. Truncated O-glycans are among the most prevalent TACAs and have been a target for mAb development for almost 40 years. Although considered poorly immunogenic 131, various Abs and Ab fragments with different affinities and specificities were developed against Tn, sTn, and TF antigens over the years.

##### Thomsen-nouveau antigen

The Tn antigen is expressed in many human carcinomas such as breast, prostate, bladder, lung, colon, and stomach. Anti-Tn mAbs have shown promising results in pre-clinical trials. However, low binding specificities and a broad tissue distribution of the Tn antigen still prevent their testing in clinical trials 131. Most scFvs against the Tn antigen were adapted from successful mAbs that show specific Tn antigen-binding properties and tumour cell growth inhibition. The Tn antigen, consisting of GalNAc, is too small for stable binding by Abs, and therefore Tn-antigens are generally recognised as two (Tn2) or three (Tn3) consecutive Tn-antigens or the Tn-antigen in combination with the peptide backbone 132. Hence, Sakai *et al*. used synthetic Tn3 for panning and screening of a phage library to generate scFvs. The generated scFvs, 4E10, and 4G2 bound Tn3 with K_D_ values of 0.37 μM and 0.14 μM, respectively 133. Other scFvs generated against Tn include 83D4-scFv 134, G2-D11 (Tn2) (K_D_ = 13.3 nM - 23.4 nM), sH1 135, MLS128 scFv (K_D_ Tn2 = 5.9 μM; K_D_ Tn3 = 1.1 μM) 136, H5, G2-H7 137, as well as 3-9 and 3-18 138. None of the described scFvs have been incorporated into Tn-targeted therapeutics. Many Tn/sTn-mucin 1 Abs have been generated that recognise not only the glycan, but a glycopeptide epitope. Efforts towards targeted therapies using such epitopes fall outside the scope of this review.

##### Sialyl-Thomsen-nouveau antigen

Sialylation of the Tn antigen to form sTn is due to overexpression of the ST6GalNAc I enzyme, and its aberrant localisation in the Golgi. Sialyl Tn has been reported to be overexpressed in breast, gastric, pancreatic, ovarian, and bladder cancer 130. The first-generation of anti-sTn mAbs was developed in the 1980s using a variety of sTn-expressing immunogens. A comparison of Abs such as B72.3, TKH2, HB-STn1, and MLS102 gave conflicting results as they differ significantly in binding among different sTn-expressing cancer tissues 139. The development of glycan arrays contributed to defining sTn specificity for mAbs as, L2A5 140, and the humanised 2G12-2B2-L0H3 141.

Several sTn-specific scFvs were adapted from B72.3, the first mAb developed against sTn 142, 143 on tumour-associated glycoprotein 72 (TAG-72) 144 using enriched membrane fractions of a breast carcinoma biopsy as immunogen 145. Muraro *et al*. generated CC49 using purified TAG-72 isolated from colon carcinoma xenografted mice as immunogen. One of the purification steps for TAG-72 included affinity purification with B72.3 146. Different formats including intact radiolabelled IgG, Fab, F(ab')2 and scFv were generated, and then compared their efficacy against colon carcinoma xenograft extracts *in vitro* and xenografted tumours *in vivo* in mice and rhesus monkeys 147. The mAb CC49 and its derived F(ab')_2_ displayed similar relative dissociation constant, whereas for the scFv and the Fab' they were 8-fold and 7.4-fold lower, respectively. The radiolabelled scFv tracer was tested successfully in a clinical trial in five patients with metastasising colorectal carcinoma using single photon emission computed tomography (SPECT) and whole-body imaging. The tracer was cleared from the blood with a biphasic clearance t_1/2_ of 30 minutes and 10.5 hours. The tumours were visualised in all patients in both primary and metastatic lesions. Although the image quality was suboptimal, the administration was safe and allowed imaging on the same day 148. In a similar preclinical study, the Colcher group generated Ab fragments derived from CC49, including scFv, diabody, Fab' and F(ab')_2_, and radiolabelled them. The scFv monomer had a 25 amino acid-helical linker, which allowed for non-covalent dimerisation to the diabody. The K_A_ for the mAb CC49, dimeric scFv and monomeric scFv were 1.7, 1.99, and 0.52 nM by Scatchard analysis and 11.4, 446, and 150 nM, respectively, by BlAcore analysis. Thereafter, pharmacokinetic, biodistribution, and tumour targeting characteristics were compared in colon as well as pancreatic cancer xenograft mice. The stable scFv dimer demonstrated a two-fold higher tumour uptake than the monomeric scFv and Fab' as well as longer retention time and, therefore, was found to have the ideal characteristics for therapeutic applications 149. Following this, the group also showed that a tetravalent CC49 scFv format improved avidity and biological half-life. The K_A_ value for the tetravalent and mAb were similar, 0.102 and 0.114 nM respectively. In addition, the K_A_ were 4-fold higher than its divalent scFv (275 nM) 150. A CC49-derived diabody was PEGylated and radiolabelled for tumour positron emission tomography (PET) imaging. The construct, PEG-AVP0458, was tested *in vivo* in colon cancer xenograft mice 151 and in clinical trials in patients with relapsed/metastatic prostate or ovarian cancer. This is the first-in-human clinical trial of a diabody, demonstrating the safety and feasibility of using diabodies for tumour imaging 152. Yang *et al*. generated a tetravalent scFv using CC49 and fused it to streptavidin for pre-targeting of Jurkat leukemia cells *in vitro* and in xenografted mice. The biotin-functionalised nanoparticles could then target the tetravalent scFv and, through it, the leukemia cells. Compared to nanoparticles only, the targeting of TAG-72 carrying leukemia cells was much higher with this strategy and presented a potential way for drug delivery to cancer cells 153. Roberge *et al*. also constructed an scFv of CC49 for therapeutic application. The V_H_ and V_L_ sequences were mutated to enhance stability, and the resulting scFv was fused to β-lactamase (BLA). BLA can be used as an enzyme to catalyse the activation of prodrugs in a treatment called antibody-dependent enzyme prodrug therapy (ADEPT) 154. The fusion protein named TAB2.5 was tested in combination with the prodrug GC-Mel in colorectal cancer xenograft mice. Cleavage of GC-Mel by BLA releases the chemotherapeutic drug melphalan. The treatment was effective, but additional optimisations of TAB2.5, the dosing regimen, and the mouse model were proposed 155. Chan *et al*. generated a different type of CC49 scFv drug conjugate by fusing the scFv to the extracellular cytotoxic domain of CD178 (FasL). The fusion boasted 30.000-fold higher cytotoxicity compared to soluble FasL *in vitro* and cured mice with intraperitoneally implanted lymphoma cells 156.

An scFv derived from humanised and mutationally improved 3E8 counterpart of CC49 (K_D_ = ~12 nM) was conjugated with IR800 to generate a tool for optical surgical navigation (OSN), which can help surgeons to resect tumours more accurately. The generated OSN agent was validated in a human orthotopic colon adenocarcinoma mouse model 157. The same group later engineered the scFv linker to optimise various properties. The best construct, 3E8.G_4_S with a low apparent K_D_ of 3.6 nM, resulted in the formation of various oligomeric states: 47% dimer, 42% trimer, and 11% tetramer. In addition, 3E8.G_4_S was shown to be an effective delivery vehicle in PET imaging of TAG-72-positive colorectal tumours in xenograft mice 158.

Anti-sTn scFvs have also been used as CARs in CAR T-cell therapy. Murad *et al*. reported a significant reduction in peritoneal ovarian tumour growth and extended overall survival of mice treated with sTn-binding second-generation CAR T-cells based on CC49. However, in early recurring tumours, reduction in expression of TAG-72 was observed, explaining decrease in T-cell persistence 159. On the other hand, results from a long-term first-generation CAR T-cell scFv huCC49 clinical trial against metastatic colorectal cancer proved disappointing. Although proven as relatively safe with minimal on target/off tumour toxicity, no clinical responses were observed 160. The reason of failure could be that the huCC49 scFv has 23- to 30-fold lower affinity compared to murine CC49, and, therefore, compromised the efficacy. A second-generation CAR T with a deimmunised version of murine CC49 scFv with high and specific tumour uptake and lower immunogenicity was generated. The CAR T-cells carried an additional binding domain specific for the tumour antigen CD47, albeit without signalling domains, to facilitate enhanced binding and avoid targeting of healthy cells. The dual CAR T-cells were effective in human ovarian xenograft mice 161.

Sharifzadeh *et al*. generated a CAR T-cell using an anti-TAG-72 Nb as binding domain fused to human CH_3_ and CH_2_ with two hinge regions. The CAR T-cell was effective in *in vitro* co-culture assays with colon adenocarcinoma and breast cancer cells. However, while 13 Nbs were initially generated and shown to bind to TAG-72 with nanomolar affinity, they were hypothesised to target different epitopes of TAG-72. Therefore, it is unclear whether the CAR T-cells bind a glycan, peptide, or glycopeptide epitope 162. In another attempt to generate sTn-targeting CAR T-cells, an scFv derived from L2A5 was used to redirect novel engineered T-cells to tumour cells in the UniCAR T strategy. The authors reported effective eradication of malignant sTn-expressing cells both *in vitro* in metastatic breast and bladder carcinoma as well as in a mouse model 163. To extend the half-life of the TM, which was initially composed of the scFv L2A5 and the UniCAR epitope only, the hinge region and Fc domain of the IgG4 Ab were added on to the UniCAR epitope. In comparison to the previous smaller-sized anti-sTn TM, the specific binding, functionality, and efficiency of the construct were then assessed on metastatic breast and bladder carcinoma *in vitro* and breast carcinoma *in vivo* assays. The construct bound with a K_D_ value of approximately 4 nM in comparison to 57 and 75 nM determined for the former construct. Efficient activation and redirection of UniCAR T-cells to cancer cells in a target-specific and TM-dependent manner as well as the secretion of proinflammatory cytokine and cell lysis were observed. The prolonged serum half-life of the anti-sTn-IgG4 TM presents a promising strategy for retargeting of UniCAR T-cells 164.

Currently, a phase I clinical trial with TAG-72 CAR T-cell therapy is being set up for patients with platinum-resistant epithelial ovarian cancer (NCT05225363).

##### Thomsen-Friedenreich antigen

The TF antigen is expressed during foetal development and in most human carcinomas. Although located in several healthy tissues, the epitope is masked by its elongated glycosylation that is truncated during malignant transformation. The TF antigen plays a critical role in metastasis through its binding to galectin on endothelial and hepatocyte cells 165.

The first TF-specific scFvs were produced via phage display selection where they focused on creating multimers based on scFvs with shorter linkers. The tetramer displayed the best binding in ELISA and SPR assays, yielding a K_D_ value of approximately 88 nM 166. They further tested the trimeric and tetrameric scFv constructs for radioimmunotargeting in a breast cancer mouse model and observed fast renal excretion and reduced kidney uptake for an scFv tetramer 167. As a validation of their previously described strategy 133, Matsumoto-Takasaki *et al*. also employed phage display screening to identify human scFvs against TF antigen. Pure scFv binds to the TF antigen in ELISA and SPR assays 168. ScFvs expressed in *Drosophila* showed specific binding. Furthermore, using STD-NMR, it was shown that the generated scFv 1E8 binds the terminal non-reducing end galactose unit of the TF-antigen 169. In a follow up study, the scFvs were expressed in *E. coli* and purified from inclusion bodies and refolded. The scFvs were active and 1E8 (K_D_ = ~83 nM) showed higher affinity than 1E6 (K_D_ = ~1.3 µM) 170. A TF-specific scFv was discovered by screening phage display libraries derived from TF-antigen immunised mice. The scFv showed binding to TF-positive leukaemia, colorectal, and gastric cancer cells, as well as inhibited adhesion of colorectal and gastric cancer cells to endothelial cells and hepatocytes *in vitro* 171.

#### *N*-glycans

The overexpression of tumour-associated *N*-glycans can serve as a marker for certain cancers, including most ovarian cancer subtypes 172. For example, the generation of human scFvs against tumour-specific glycoforms containing bisecting *N*-glycans of the ECM protein Periostin via yeast display led to functional fragments, of which one (scFvC9) was validated for its tumour specificity in ovarian cancer cells *in vitro* and xenografted in mice 173. Such *N*-glycosylation-specific Ab fragments may be novel agents in cancer diagnosis and therapy. Another *N*-linked TACA is mannotriose, a trisaccharide composed of the three mannose moieties on the core base of canonical *N*-glycans. In normal cells, core mannose residues are masked by terminal glycosylation, whereas in cancer cells, they can become exposed due to aberrant glycosylation 174. Therefore, scFvs against mannotriose were developed as a putative therapeutic tool and tested on breast cancer cells along with anti-Tn scFv 136 *in vitro*. However, the scFvs (1A4, 1G4 and 5A3 with K_D_ values of 2.5, 1.8 and 3.8 µM, respectively) did not inhibit breast cancer cell growth, and the potential application for cancer therapy still needs to be developed 175.

### Proteoglycans

Proteoglycans are major components of the extracellular matrix (ECM) in animal tissues and are composed of a protein part and a glycosaminoglycan (GAG) moiety. GAGs are long linear repeats of disaccharide units that consist mainly of a uronic acid and an amino sugar. An important structural feature of GAGs is the extensive *N*- and *O*-sulfation, which plays vital roles in cell-cell and cell-ECM interactions due to its negative charge. Consequently, alterations in sulfation patterns often have adverse effects and are associated with various disorders, including several cancer types 176. Based on the identity of the disaccharide repeats, GAGs are divided into four groups: heparin/heparan sulfate, chondroitin/dermatan sulfate, keratan sulfate, and hyaluronan. Despite the important roles GAGs play in numerous signalling pathways, Abs and Ab fragments targeting specific GAG epitopes are scarce. This is mainly due to the enormous complexity of the sulfation pattern along the GAGs backbone chain, referred to as the “sulfation code”. The high structural complexity of sulfate groups on the disaccharide repeats prevents the development of molecular tools that target specific GAG epitopes. Therefore, nearly all anti-GAG Abs lack selectivity among the various structures of GAGs and, hence, have broader targets, such as heparan sulfate or chondroitin sulfate. Another limiting factor is access to pure, structurally defined GAGs oligosaccharides for Ab generation and characterisation, as isolation from natural sources is highly challenging, and chemical synthesis is generally slow, costly, and low yielding. Therefore, the importance of GAGs demands new, sulfation-specific Abs that would help to better understand GAGs' biology and the structure-to function of specific sulfation patterns 177.

#### Heparan sulfate

A predominant class of proteoglycans is heparan sulfates (HS), which are involved in a variety of biological processes, for instance, growth factor signalling and cell adhesion 177. Changes in the degree and pattern of sulfation of HS are associated with breast, ovarian, colorectal, and gastric cancers, among others. It has been suggested as a biomarker for breast cancer 176. Aberrant sulfation of HS has been shown to negatively affect the survival of patients with head and neck squamous cell carcinoma 178. Due to the low immunogenicity of HS, conventional methods to generate anti-HS Abs are very challenging. However, that problem has been overcome by generating HS-specific Ab fragments using human phage display library 179. Kuppevelt *et al.* generated scFvs using a synthetic human phage display library against HS from bovine kidney and became the first group to generate scFvs against polysaccharides 180. The obtained scFvs had K_D_ values of 0.12 and 0.15 µM. The anti-HS scFv library was used to study HS distribution in muscle basal lamina of various species (including human, mouse, and rat) and found the scFv binding to be strongly sulfation dependent. HS differences were found between the neural, synaptic, and extrasynaptic basal laminae, and changes in distribution during muscle development were time- and region-dependent both *in vitro (*mouse-derived skeletal muscle and mutant Chinese hamster ovary cells) and *in vivo (mouse and rat model)*. The study underlines the importance of HS in skeleton muscular and neural development 181. Further, by using HS from synthetic and native sources and anti-HS scFvs that bind distinct HS sulfation patterns, the group identified 15 varying HS domains with specific tissue distribution, indicating a tightly regulated topological distribution 182. Anti-heparin scFvs were also identified and characterised as well as investigated for inhibition of heparin anticoagulant effect. The scFvs could bind heparin on mast cells in human lung cryosection. Some scFvs exclusively bound to heparin, while others also bound to structurally related HS and chondroitin sulfate 183. Notably, most Ab fragments against HS exhibit a similar affinity to heparin. Because medicinal heparin can have various severe side effects, including heparin-induced thrombocytopenia, agents are needed to block the anticoagulant effect if necessary 184. Therefore, scFvs providing the required inhibitory effect on anticoagulation could be promising candidates 183. Even though defined HS structures were used in the studies, the oligosaccharide sequence was largely unknown. Therefore, the group identified a specific scFv (MW3G3) against acharan sulfate, which is a HS-similar snail GAG and has a repeating disaccharide structure of α-d-*N*-acetylglucosaminyl-2-*O*-sulfo-α-l-iduronic acid (GlcNAc-IdoA2S) residues. The scFv was then used to identify HS oligosaccharides containing the disaccharide repeats and study their distribution in rat organs 185. Using another scFv (RB4CD12) that recognises highly sulfated domains (6S-domains) of HS, it could be shown that HS S-domains play a significant role in lung and ovarian cancer progression 186-188. Recently, one of the well characterised scFvs from the initial synthetic phage display library, HS4C3, which binds strongly to the 3-*O*-sulfated motif in HS and weakly to any *N*-sulfated, 2-*O-* and 6-*O*-sulfated hexa- to octasaccharide fragment 189, was also used as a template, containing a heparan-binding consensus site in CDR3 to establish another scFv library with a large number of scFvs against HS only differing in the consensus region 190.

Sulfation-specific scFvs, D4A4 and D6B10, were obtained against syndecan-1, an HS proteoglycan associated with multiple myeloma (MM) cells. Both scFvs pulled down syndecan-1 from MM cell lysates as well as competed for binding to cells. D4A4 and D6B10 scFvs may provide help reading changes in sulfation patterns of HS during myeloma tumour progression 191. Another scFv, HS20, was selected from a phage display library against glypican-3, which is often highly expressed in hepatocellular carcinoma and correlates with poor prognosis. The scFv was, however, converted to a full IgG that showed binding to a Wnt binding domain in the HS structure and inhibited Wnt signalling, suppressing hepatocellular carcinoma cell growth *in vitro* and in xenografted mice 192.

#### Chondroitin and Dermatan sulfate

Chondroitin sulfate (CS) plays an essential role in skeletal development and is responsible for 67-97% of total GAG content of bone. As coreceptors in various signalling cascades, the sulfation degree and pattern can greatly affect skeletal development. The sulfation changes can also be associated with cancers such as breast, ovarian, colorectal, prostate, and gastric cancers, among others 176. Therefore, investigating CS-specific Ab fragments is highly relevant regarding both cancer diagnosis and therapy. With the phage display technique that had previously been used for HS 180, Smetsers *et al.* used a synthetic human scFv library for the selection of Ab fragments against CS. The scFvs IO3D9, IO3H10, and IO3H12 possess high specificity for CS, whereas IO4C2 also shows cross-reactivity with heparin. While all reacted to CS-A, CS-C, and CS-E, IO3D9 showed higher specificity towards CS-E, which is highly sulfated compared to CS-A and CS-C. Expression and localisation of CS were studied on rat organs and melanoma metastases *in situ*. These may be valuable tools in detecting alterations in CS patterns in both healthy and malignant tissues 193. E-units on CS-E and dermatan sulfate (DS) are involved in metastasis of Lewis lung carcinoma cells. The scFv GD3G7 recognises E-units, GlcAβ1-3GalNAc(4S,6S), where 4S and 6S stand for 4-*O*- and 6-*O*-sulfate, respectively. Using GD3G7, it was shown that the presence of the E-unit was crucial for the metastatic potential and that the addition of GD3G7 and CS-E decasaccharide fraction (minimal epitope of the scFv) could strongly inhibit metastasis 194. GD3G7 was also used to test 148 ovarian tumours including benign and malignant for the presence of 4,6-disulfated CS motifs, potentially an ovarian cancer biomarker. 4,6-disulfated CS had significantly higher expression in malignant tumour, and its expression correlated with serous subtype, high tumour grade, advanced FIGO-stage and high CA-125 levels 195. The scFv GD3A10 was selected in the biopanning against embryonic GAGs as a source of cancer antigen. It specifically targeted the GAG moieties of CS associated with ovarian cancer 196. In the next study, the binding specificity of scFvs GD3A11 to malignant ovarian tumours was confirmed in large-scale studies (N = 359) on human patient tissues. Unlike with GD3A10, the expression of highly sulfated CS showed no correlation to tumour grade, FIGO stage, and the use chemotherapy. For aggressive ovarian cancer, however, high expression independently predicted poor prognosis 197.

The two scFvs, GD3A12 and LKN1, were also generated against 4/2,4 di-*O*-sulfated DS, which was found to be expressed in rat thymus and spleen. In ovarian tumours, DS expression was high in the stromal parts, and occasionally on tumour cells. These scFvs may help study the function, expression, and localisation of DS in healthy tissue as well as cancer 198, 199. GAGs and their sulfation moieties play an important role in the ECM and aberrant alterations including increased sulfation of CS have been proposed as potential cancer biomarkers 176.

## Conclusion

Due to their boasted thermo- and chemostability as well as their small size, Ab fragments have been investigated as therapeutics against several diseases targeting different glycan antigens. Access to cryptic epitopes that are out of reach for conventional Abs, including the heavily concentrated tumour microenvironment, as well as high stability of small fragments are major advantages. Moreover, the lack of a Fc portion reduces the risk of side effects as immune effectors such as the complement-dependent cytotoxicity will not be activated.

Ab fragments can be manipulated into being functional in many ways. By selecting a specific format, the pharmacokinetics of the targeting moiety can be adapted for the application. In general, increasing size leads to increased half-life. For imaging purposes, the half-life should be short, whereas it should be longer for therapeutic purposes to exert biological effects. Increasing size decreases tumour penetration. Hence, each fragment has advantages and disadvantages regarding different applications. Prolonging their circulation half-life can be accomplished by PEGylation, fusion with an albumin-binding Ab fragment or multimerisation 200. Increasing valency can aid in improving the overall affinity for the targets through avidity.

TACA-targeting scFvs have been researched the most compared to any other Ab fragment. The widespread applications such as imaging agents, drug conjugates, BiTES, and CAR-T cells allow for easy multimerisation in the form of taFv and diabodies. The small Ab fragments targeting TACAs that reached clinical trials mostly represent scFvs incorporated in CAR T cells for Le^Y^, sTn, and particularly for GD2. Other treatment modalities still face problems due to toxicity or immunogenicity. Nonetheless, small Ab fragments are particularly suitable for tumour imaging, illustrated by numerous reports of pharmacokinetic properties of fragments in murine xenograft models. The sTn-specific PEGylated and radiolabelled diabody PEG-AVP0458 was the first diabody to reach clinical trials 152. It was successfully used in PET imaging, emphasising the suitability of using recombinant anti-TACA Ab fragments in cancer diagnosis. The Nb format has been gaining increasing interest since it is more stable and easier to produce and functionalise. Recently published Nbs against Globo-H 41, and GD2 (patent ID: CN110551218B) indicate the ability to produce even smaller antibody fragments targeting TACAs to further innovate cancer diagnosis and therapy.

## Figures and Tables

**Table 1:** GD2-targeting CAR T-cells in clinical trials.

Identifier	Phase	scFv	Generation	Disease	Age	(Estimated) completion
NCT01460901	I	14.G2a	1	Neuroblastoma	1.5-17	January 2015
NCT02107963	I	14.G2a	3	GD2+ solid tumour	1-35	January 2017
NCT02919046	I/II	14.G2a	3	Neuroblastoma	1-14	September 2020
NCT02992210	I/II		4	GD2+ solid tumour	1-65	December 2020
NCT03356795	I/II			Cervical cancer	18-70	December 2020
NCT02761915	I	KM8138	2	Neuroblastoma	1+	August 2021
NCT02173093	I		4	Neuroblastoma	1-29	December 2022
NCT02765243	I	Hu3F8	4	Neuroblastoma	1-14	December 2022
NCT03423992	I			Glioma	18-70	January 2023
NCT00085930	I	14.G2a	1	Neuroblastoma	< 21	June 2023
NCT04637503	I/II		4	Neuroblastoma	1-65	December 2023
NCT03356782	I/II	hu3F8	4	Sarcoma, osteoid Sarcoma and Ewing Sarcoma	1-75	December 2023
NCT04433221	I/II			Sarcoma Osteoid Sarcoma Ewing Sarcoma	1-75	December 2023
NCT04430595	I/II		4	Breast cancer	18-75	December 2023
NCT04429438	I/II		4	B cell lymphoma	0.5-75	December 2023
NCT04539366	I	14.G2a	3	Neuroblastoma and osteosarcoma	<35	August 2024
NCT05438368	I/II		(bispecific)	GD2+ and CD70+ solid tumour	1-75	June 2026
NCT05437315	I/II		4 (bispecific)	GD2+ and PSMA+ solid tumour	1-75	June 2026
NCT05437328	I/II		4 (bispecific)	GD2+ and CD56+ solid tumour	1-75	June 2026
NCT03373097	I/II	14.G2a	3	GD2+ solid tumour	1-25	December 2027
NCT01822652	I	14.G2a	3	Neuroblastoma	All	October 2030
NCT03294954	I	14.G2a	2 (NK cells)	Neuroblastoma	1-21	August 2034
NCT01953900	I	14.G2a	3	Neuroblastoma and sarcoma	All	October 2034
NCT05298995	I		3	GD2+ CNS tumour	0.5-30	May 2037
NCT03635632	I		4	GD2+ solid tumour	1-74	December 2038
NCT04099797	I	14.G2a	4	GD2+ brain tumours	1-21	February 2038
NCT03721068	I	14.G2a	4	Neuroblastoma and osteosarcoma	1.5-18	June 2039
NCT04196413	I	14.G2a		Diffuse intrinsic pontine glioma and spinal diffuse glioma	2-30	July 2042
